# Large language models identify immigration attitudes in online discourse regardless of language

**DOI:** 10.1038/s41598-026-52167-6

**Published:** 2026-05-20

**Authors:** Andrea Nasuto, Stefano Iacus, Francisco Rowe, Devika Jain

**Affiliations:** 1 Center for Geographic Analysis, Harvard University, Cambridge, USA; 2 University of Liverpool, Liverpool, UK; 3 European Commission, Joint Research Centre, Seville, Spain

**Keywords:** Language and linguistics, Language and linguistics, Mathematics and computing

## Abstract

Large language models (LLMs) offer new opportunities for scalable analysis of online discourse. Yet their use in multilingual social science research remains constrained by model size, cost and linguistic bias. We develop a lightweight, open-source LLM framework using fine-tuned LLaMA 3.2–3B models to classify immigration-related tweets across 13 languages. Unlike prior work relying on BERT-style models or translation pipelines, we combine topic classification with stance detection and demonstrate that LLMs fine-tuned in just one or two languages can generalize topic understanding to unseen languages. Capturing ideological nuance, however, benefits from multilingual fine-tuning. Our approach corrects pretraining biases with minimal data from under-represented languages and avoids reliance on proprietary systems. With 19–96$\times$ faster inference and up to 2,017$\times$ cost savings compared to commercial LLMs, our method supports real-time analysis of billions of tweets. This scale-first framework enables inclusive, reproducible research on public attitudes across linguistic and cultural contexts.

**Supplementary Information:**

The online version contains supplementary material available at 10.1038/s41598-026-52167-6.

## Introduction

The widespread availability of generative AI, particularly large language models (LLMs), is transforming scientific research and more generally human practices. These models are more accessible, easy to use and deployed across a range of text-based tasks in all academic fields. In the social sciences, LLMs have been used to analyze political speech^1^, finance sentiment^2^, social media opinions on immigration  ^3^, enhance systematic literature reviews^4^ and improve text classification^5^.

Despite the growing adoption of LLMs, important gaps remain in our understanding of how fine tuned LLMs can classify text across multiple languages while remaining cost effective and environmentally sustainable at scale. Most prior work relies on encoder based BERT family models rather than decoder only LLMs, evaluates performance on benchmark datasets without considering the challenge of deploying models across trillions of tokens and focuses primarily on hate speech classification instead of jointly modeling topic relevance and nuanced stances toward immigration on online social media, which has become a key societal challenge across the globe.

In this paper, we study whether a relatively small, open source decoder-only LLM can be fine tuned to classify a culturally rich and politically salient issue, such as immigration across multiple languages using different highly efficient and environmental-friendly fine-tuned LLMs. We also assess whether that model can generalize both topic and stance classification across many languages while remaining financially and computationally efficient to be deployed at scale. We compare several fine tuning setups, including models trained on a single language, on two languages, and on a multilanguage mix, and evaluate how well each configuration transfers to unseen languages. To benchmark this approach, we also fine tune a multilingual encoder-only BERT (mBERT) model on the same training data and evaluate its performance alongside LLaMA 3.2 3B. This design allows us to directly compare encoder-only and decoder-only architectures in multilanguage topic and stance classification. This design enables a controlled comparison between encoder-only (mBERT) and decoder-only (LLaMA) architectures in multilanguage topic and stance classification

Over the past decade, the underlying architectures and scales of these models have changed substantially, giving rise to two paradigms that differ in their suitability for multilingual stance classification. Early transformer based systems such as BERT were encoder-only models with roughly 110M to 340M parameters, pretrained on about 3.3B tokens^6^. Legacy encoder based architectures in the BERT family, including multilingual BERT and RoBERTa, have been the workhorse of most prior cross-lingual classification research because they are open source and easy to fine-tune and their relatively lightweight size makes them appealing for large scale deployment^7, 8^. However, these models underperform on semantically demanding and knowledge-intensive classification tasks that involve implicit meaning, coded expressions and obfuscated hostility^9^, all of which strongly characterize immigration discourse on social media. The current generation of decoder-only LLMs, including OpenAI’s GPT-style models and Meta’s Llama family, addresses these limitations by scaling both parameters and pretraining data by orders of magnitude. For example, Llama 3.2 3B has about 3B parameters, about 10 times larger than BERT and was pretrained on 15.6 trillion tokens^10^, enabling emergent capabilities including general purpose multilingual reasoning that can follow instructions and handle complex, context and knowledge intensive tasks^9, 11^. Empirical evidence supports this advantage:^12^ show that a fine-tuned LLaMA 3.1 8B model outclasses BERT based models in detecting hate speech in a culturally rich and multilingual setting with coded language. Consequently, findings and properties established in prior work on BERT family encoders should not be assumed to extend to modern instruction tuned LLMs and while BERT family models remain useful points of comparison, a decoder-only LLM such as LLaMA 3.2 with 3 billion parameters is a more appropriate backbone for the high context, multilingual stance classification problem we study here.

The transformative potential of LLMs does not come only from scaling up pretraining data or increasing the number of parameters, but from their ability to be adapted through fine-tuning. Fine-tuning allows turning a general-purpose foundation model into a task-specific system using domain-relevant labelled data, improving task performance and encoding domain-specific knowledge without the need to retrain a model from scratch.

Social science applications could in principle leverage these growing capabilities to study large scale text corpora, but as datasets grow to hundreds of billions or trillions of tokens, running LLMs at inference scale raises substantial computational, financial and environmental costs^13^. Running inference with large LLMs or commercial APIs on such volumes can quickly exceed typical academic budgets and has nontrivial energy demands, which constrains the kinds of models that can be used in practice.

Existing work on fine tuned LLM classifiers has therefore tended to operate either on small curated benchmarks or synthetic tasks. On the one hand, encoder based BERT family models are efficient and more sustainable but underperform decoder only LLMs on multilingual, knowledge intensive and reasoning heavy tasks^9^. On the other hand, when decoder only models are used, they are often very large (e.g. Llama 70B) or accessed via commercial APIs such as ChatGPT, which makes it difficult to assess whether these setups can be deployed cost effectively at scale for social science workflows. Both paradigms present limitations that have constrained the scope of prior research. Past studies typically focus on binary labels^7^ and on LLMs fine tuned in a single high resource language such as English, and do not test whether a single fine tuned model can jointly handle topic detection and multi class stance classification across many languages^12^.

In multilingual classification tasks, a straightforward strategy is to fine tune a separate LLM for each language in the dataset. Yet fine tuning on many languages is costly in terms of time, computational resources and labeled data^14^). One common workaround is to translate all content into a single language, typically English and fine tune a model only on that language. However, this approach is known to introduce translation biases that harm classification performance and can amplify cultural and gender biases^10, 15, 16, 17^. While translating with state of the art LLM based systems may be feasible for small or medium sized corpora, applying this strategy to datasets on the order of trillions of tokens would require either substantial computational infrastructure or financial resources in the millions of dollars when using commercial APIs (see Table 8 in the SI Appendix). This makes naive translate then classify pipelines impractical for large scale social media analysis.

Pretraining data are known to shape a model’s general language knowledge^18, 19^, and heavily imbalanced exposure can generate systematic performance gaps across languages in classification tasks^20^. Contemporary LLMs are, in fact, pretrained predominantly on English data^10^. This imbalance extends beyond language to culture, as LLMs have been shown to align more closely with the values of English speaking and Protestant European societies^21^. Such biases may influence how immigration is represented by the model and may also reduce its ability to identify immigration related content in non English languages^22^.

These open questions are especially salient in the case of online public debate on immigration, a globally salient and ideologically charged topic that intersects with debates over national identity, labor markets, human rights and security^23^. Its discussion is shaped by context specific cultural and geographic dynamics and it frequently provokes polarized public opinion, including physical violence. Social media platforms have become central arenas for immigration discourse, where anti immigration narratives, misinformation and hate speech often circulate^3^. These platforms not only reflect public attitudes but can exacerbate divisions and amplify hostility toward migrants^24^. This complex communicative landscape makes immigration an ideal testbed for evaluating how LLMs generalize their classification capabilities across languages. Yet little is known about the cross lingual properties of fine tuned LLMs in understanding such culturally rich and politically charged content.

Most existing work classifying social media content has focused on using LLM for hate speech detection^7^. These problems are typically framed as a binary decision between hateful and non hateful content. By contrast, our task requires first deciding whether a tweet is related or unrelated to immigration and if this is the case, our model seek to classify if posts express a pro-immigration, anti-immigration or neutral stance. This narrower focus on immigration makes the task more challenging and realistic than identifying hate speech. Hate speech is a broad umbrella category that can cover misogyny, racism, homophobia and many other target concepts. Immigration expressions include subtle policy disagreements, coded language and context dependent cultural references. Although some anti-immigration messages clearly overlap with hate speech, our labels are not binary by also capturing neutral and pro-immigration stances that are typically ignored in standard hate speech classification. As such, the task of classifying immigration attitudes across languages is both a methodological challenge and a socially essential goal. Scalable, accurate classification tools are urgently needed to understand global digital discourse on immigration in real time. Beyond hate speech, we recognize that past work has also examined the cross-lingual and cross-cultural properties of LLMs.^25^ focuses on incorporating cultural differences into LLMs and measuring culture specific behavior across groups rather than multilingual classification tasks generability.^26^ evaluates multilingual generative AI systems across a wide range of languages and tasks but without fine-tuning and closed-access commercial models.^27^ use LLMs for cross lingual data augmentation in commonsense reasoning tasks not classification.^28^ fine-tuned model to classify immigration-related content on a benchmark and only for Spanish and based on encoder models like multilanguage BERT and BETO. We are not aware of any study examining the multilingual properties of fine-tuned LLMs tested in a real case scenario with hundreds of billions of tokens to be classified, not a small benchmark dataset. Table 1 in the SI summarizes the main strands of previous literature and compares them in terms of findings, strengths, limitations and remaining gaps.

These gaps lead to test four hypothesis. Specifically:

1. We test whether a lightweight open-source decoder-only LLM fine-tuned on one or two languages can accurately classify tweets in languages it has never seen during fine-tuning as related or unrelated to immigration (topic classification) and whether its performance matches or exceeds that of a model fine-tuned on data that include the target languages.
2. We test whether a lightweight open-source decoder-only LLM fine-tuned on one or two languages can accurately classify immigration-related tweets in languages it has never seen during fine-tuning as pro immigration, anti immigration or neutral (stance classification) and whether its performance matches or exceeds that of a model fine-tuned on data that include the target languages.
3. We test whether imbalances in language representation during pretraining generate systematic accuracy gaps across languages in both topic classification and stance classification, and whether targeted fine-tuning can reduce these biases even with very small amounts of additional training data.
4. We assess whether a lightweight open-source LLM that has been fine-tuned and quantized can deliver substantial financial and environmental advantages over commercial LLMs when deployed at very large inference scale.

To test how well language models generalize across languages under these constraints, we fine tune the lightweight open source Meta LLaMA 3.2 model with 3 billion parameters to classify immigration related tweets using real, human annotated data. The human annotated dataset is a sample of geolocated tweets in 12 different languages and geographies extracted from the Harvard GeoTweet Archive 2.0^29^, annotated using a standardized codebook. We split the dataset into training and test sets: the training set is used to fine tune our models and the test set is used to evaluate performance on unseen data. We experiment with 4 fine tuned models: (i) a monolingual model trained on English data only; (ii) a monolingual model trained on Spanish data only; (iii) a bilingual model trained on a combined dataset that includes both the English and Spanish data used in models 1 and 2; and (iv) a multilingual model trained on this same English Spanish dataset along with human annotated tweets in 9 additional languages, covering a total of 11 training languages and 13 evaluation languages. This experimental setup allows us to assess whether exposure to multiple languages during fine tuning leads to higher classification accuracy or if comparable results can be achieved using fewer languages. We evaluate each model on languages that were excluded from its training set, focusing on a 4 way classification task: determining whether a tweet is (i) unrelated to immigration, or expresses a (ii) neutral, (iii) pro immigration or (iv) anti immigration stance. To test the effectiveness of translation as an alternative strategy, we machine translated and then classified English translations of non English tweets. All models are trained on a near zero emission GPU cluster at Harvard and deployed in quantized format for computational efficiency. This design enables us to assess whether task specific fine tuning on one or more languages can teach LLMs to classify content in other unseen languages, particularly those underrepresented in the LLM pretraining, while remaining viable at the scale required by the GeoTweet Archive.

Our findings show that a bilingual model fine tuned in English and Spanish can reliably detect whether a tweet is about immigration, even when the tweet is written in a language the model has never seen during fine tuning. This suggests that the model learns a general representation of the topic that transfers across languages. However, the multilingual model trained on 12 languages performs better when the task requires identifying stance, that is whether a tweet expresses a pro immigration, anti immigration or neutral position. Exposure to diverse linguistic and cultural contexts during fine tuning appears to enhance the LLM’s ability to capture nuanced ideological content. Importantly, these results are achieved without relying on high cost machine translation, synthetic prompts or complex multilingual pipelines. In contrast, prior studies often construct multilingual datasets by machine translating entire datasets^30^, mix multiple languages within prompts^31^, or chain translations of both prompts and responses^32^. Our method uses real, human annotated data and standard supervised fine tuning. This design provides a transparent and reproducible alternative that is more practical for real world multilingual applications.

To demonstrate the scalability of our approach, we estimate that our fine tuned model could classify the entire Harvard GeoTweet Archive 2.0, which contains more than 10 billion tweets, for about 2,900 dollars with virtually zero additional carbon emissions, compared to proprietary alternatives that would cost between 316,000 and 17.7 million dollars while generating hundreds to thousands of tonnes of CO_2_. Moreover, our model operates at 3,854 tokens per second, between 36 and 168 times faster than comparable commercial alternatives.

To our knowledge, this is one of the first applied studies in computational social science to systematically evaluate whether open source LLMs fine tuned in one or two languages can generalize to classify both the topic and stance of text written in 13 languages, including several languages that are unseen during fine tuning for each model, under realistic computational and budget constraints. It offers direct empirical evidence that LLMs can learn to perform specific tasks effectively across languages, without relying on familiar linguistic patterns seen during training. This opens the door to efficient multilingual classification without the need to fine tune a separate model for each language. This opportunity is especially relevant given that fine tuning across many languages typically requires substantial computational resources, annotated data and technical capacity that are not always available. Our study offers concrete evidence for how socially relevant tasks, such as monitoring the online debate on immigration on social media, can be achieved through scalable, cross lingual AI systems that bridge LLMs with real world social science research.

Our study makes two significant contributions. First, we contribute novel and robust empirical evidence of the ability of a small decoder only LLM to identify the semantic intent of messages in multiple languages that it has not been fine tuned on. We show that LLMs can accurately classify migration related content across multiple languages following exposure to text in one or two languages via fine tuning. We present evidence of the generalizable potential of LLMs through deploying learned knowledge from a single language to a range of multiple languages at two classification tasks: (i) identifying migration related content; and (ii) identifying anti and pro migration stances in the text.

Second, we make a methodological contribution. We develop a cost efficient and scalable framework for the deployment of open source LLMs for large scale text classification tasks^13^. We demonstrate this approach by fine tuning a lightweight, open source large language model (LLaMA 3.2 3B) using Low Rank Adaptation (LoRA)^33^, an efficient method that significantly reduces the number of trainable parameters. The fine tuned model is then quantized to 4 bit precision using the GPT Generated Unified Format (GGUF), which enables efficient deployment on resource constrained hardware. This quantization step reduces the overall model size by about 50 percent compared to the original model, while preserving high classification accuracy and substantially lowering computational requirements. It efficiently optimizes LLMs, resulting in low requirements for computing infrastructure and energy and in principle enabling LLM deployment even on small CPU based consumer machines. As such, our proposed approach makes LLM deployment more accessible and environmentally sustainable. Such gains represent important evidence for the effective future use of LLMs and address concerns about the high environmental cost, elevated energy consumption and unequal access to LLMs. Taken together, these contributions have the potential to augment the use of LLMs in the social sciences by fine tuning models in a limited number of languages and deploying them to execute analysis across multiple languages and geographies. Such advances can fill gaps in our understanding of human and social behaviors across different cultural and linguistic settings.

## Results

This section presents the results of our evaluation task: assessing whether fine-tuning a lightweight open-source model, LLaMA 3.2 3B, on one or more languages enables it to learn the concept of immigration-related content in a way that generalizes across multiple languages. Specifically, we test the model’s ability to identify whether a tweet is about immigration and, if so, determine the stance it expresses (“pro-immigration”, “anti-immigration”, or “neutral”) across both seen and unseen languages.

Our findings point to 4 main conclusions. First, topic-level understanding transfers across languages with minimal fine-tuning: a model trained on only English and Spanish can reliably identify whether a tweet is about immigration even in languages it has never seen. Second, stance detection, meaning the ability to distinguish pro-immigration, anti-immigration and neutral positions, is more language-dependent and benefits substantially from multilingual fine-tuning. The model trained on 12 languages consistently outperforms monolingual and bilingual variants on this subtler task. Third, pretraining language imbalances are associated with accuracy gaps across languages, but targeted multilingual fine-tuning can largely correct these biases even with very small amounts of training data in under-represented languages. Fourth, machine translation degrades classification accuracy relative to direct inference on native-language text, even when translation quality is rated as good, reinforcing the case for training on original content. Together, these results demonstrate a practical and scalable path to multilingual classification that avoids costly translation pipelines and proprietary APIs.

**Fig. 1:**
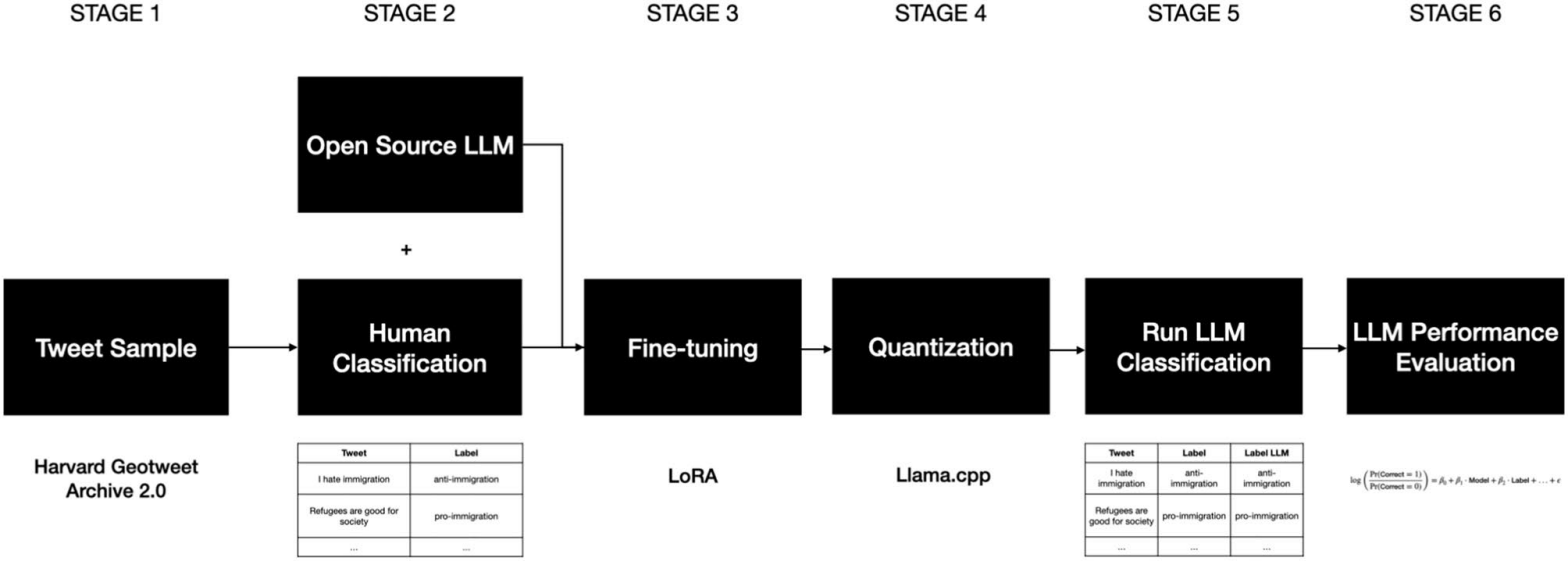
Overview of the workflow used in this study.

Figure 1 provides an overview of the workflow used in this study, which consists of six main steps: collection of a sample of tweets, human annotation, LLM fine-tuning and subsequent quantization, tweet classification (both topic and stance) and several logistic regression models to evaluate the classification results across multiple factors.

Firstly, we extract a sample of tweets in 13 languages from the Harvard Geotweet Archive 2.0, a collection of 10 billion geolocated tweets. These tweets are selected using immigration-related search terms (see Table 2 in SI for details). Each tweet is subsequently labeled by human annotators. Annotators first determined whether a tweet was related or unrelated to immigration. If deemed relevant, the tweet was further classified as expressing a positive (pro-immigration), negative (anti-immigration), or neutral stance towards immigration. Annotation guidelines were iteratively refined to ensure consistency across languages and annotators. Non-English tweets were translated using a LLaMA 3.2 Instruct - 3B quantized 4bit model. Table  4 in the SI Appendix reports the number of tweets annotated per language, showing the linguistic diversity and balance across data used for model training and evaluation.

We developed four fine-tuned LLMs, each trained on a human-annotated data: (1) a monolingual English model, (2) a monolingual Spanish model, (3) a bilingual English–Spanish model and (4) a multilingual model trained on twelve languages. These four models allow us to compare how fine-tuning on different languages affects generalization to unseen languages and classification accuracy. The monolingual English model is trained using only the English labeled tweets, similarly the Spanish model is trained on Spanish labeled tweets. The English–Spanish model is trained on the combined English and Spanish datasets used in the respective monolingual models. The Multilingual model is trained on this same English and Spanish data, along with additional samples in Arabic, French, German, Hindi, Hungarian, Indonesian, Italian, Polish, Portuguese and Turkish. The Korean sample is not included in the training of any of the four LLMs. We also fine-tuned Multilanguage BERT (mBERT) models under the same experimental configurations as the LLaMA models, including English-only, English-Spanish, Spanish-only and Multilanguage setups, to benchmark performance across model architectures. We report weighted precision, recall and F1 scores for both LLaMA and mBERT models across all classification tasks in Table 7 in the SI Appendix. Across all configurations, the fine-tuned LLaMA models consistently achieve higher performance than their mBERT counterparts. This suggests that the larger pretraining scale and decoder-only architecture of LLaMA models provide an advantage in capturing the semantic and contextual complexity of immigration-related discourse.

We estimated four logistic regression models to analyze data from all four fine-tuned LLMs and assess the factors associated with the probability that a fine-tuned language model correctly classifies the standing towards of immigration of the tweets. The dependent variable is binary, indicating whether the model’s predicted label matches a human-annotated ground truth. In all model specifications, the baseline corresponds to a model fine tuned on English language tweets, applied to untranslated input, with the tweet labeled as Neutral in terms of immigration stance by the human annotators, written in English and belonging to the training set. The regression intercept reflects the classification accuracy under this baseline condition. All other coefficient estimates represent deviations from this reference point as log-odds. Positive coefficients indicate a higher likelihood of correct classification compared with this baseline, while negative coefficients indicate a lower likelihood. Larger absolute values reflect stronger differences from the baseline.

**Fig. 2:**
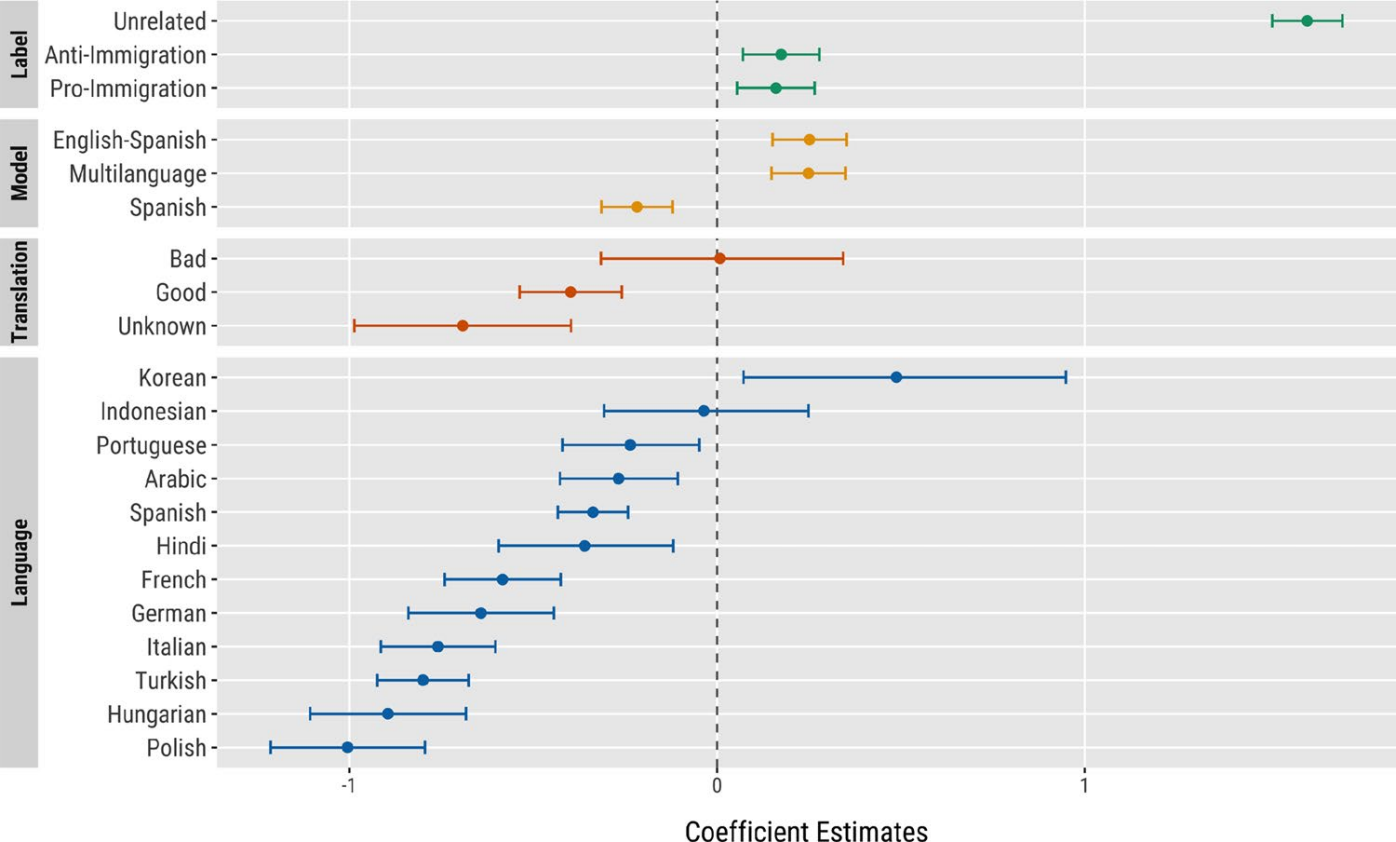
Estimated coefficients (log-odds) from the baseline logistic regression model. The figure displays point estimates and 95% confidence intervals for the main effects of *Model* type, tweet *Language*, *Translation* quality and *Label*. The model estimated the probability that a fine-tuned language model correctly classifies a tweet’s stance toward immigration. Coefficients are expressed in log-odds and grouped by covariate type. Our baseline model is the one fine-tuned exclusively on English data, evaluated on untranslated tweets labeled “Neutral“ from the “Train“ set. Estimates are derived from Model 1, which includes no interaction terms (Eq. 3 in the SI Appendix). The log-odds coefficient for *Train/Test* is excluded from the figure.

Figure 2 presents the results from a baseline logistic regression model (Model 1) predicting the likelihood of correct tweet classification, expressed in log-odds, as a function of model type, label category, input language, translation quality and whether the tweet is part of the test or training dataset split. The full coefficient estimates are reported in Table 10 in the SI Appendix.

Tweets labeled as Unrelated are significantly easier to classify compared to the baseline category Neutral ($\beta = 1.579$, $p < 2 \times 10^{-16}$), followed by tweets expressing pro-immigration ($\beta = 0.222$, $p < 3.7 \times 10^{-8}$) and anti-immigration views ($\beta = 0.215$, $p < 1.1 \times 10^{-7}$). There is notable variation across languages. Tweets written in Polish ($\beta = -1.034$, $p < 2 \times 10^{-16}$), Hungarian ($\beta = -0.865$, $p < 2 \times 10^{-16}$) and Italian ($\beta = -0.710$, $p < 2 \times 10^{-16}$) are substantially harder to classify than English tweets, which serve as the reference category.

The bilingual English-Spanish model ($\beta = 0.280$, $p < 3.3 \times 10^{-13}$) and the Multilingual model ($\beta = 0.263$, $p < 6.4 \times 10^{-12}$) both outperform the English-only baseline, highlighting the benefits of multilingual exposure during fine-tuning as well as a larger size of training dataset. On the other hand, the Spanish-only model performs significantly worse ($\beta = -0.215$, $p < 5 \times 10^{-8}$) compared to the English model baseline.

Translation significantly reduces classification accuracy, regardless of assessed quality. Tweets that were machine-translated from other languages into English tend to be harder to classify correctly than their original, untranslated counterparts. Even tweets marked as Good translations show a significant decrease in the ease of classification ($\beta = -0.381$, $p < 3.9 \times 10^{-8}$), while Unknown translations perform worse still ($\beta = -0.680$, $p < 1.2 \times 10^{-6}$). Translations classified as Bad do not show a statistically significant effect, likely due to limited sample size. Translation likely introduces semantic misalignments as seen in previous research^15^. For example, in the Italian immigration discourse, the term *clandestini* may be translated as *migrants* or *undocumented migrants*, muting the pejorative meaning of the original term. As a result, translated tweets become semantically more ambiguous and harder to classify accurately as anti-immigration, pro-immigration, or neutral.

### Fine-tuning teaches the topic regardless of language

We investigated whether the impact of fine-tuning varies not only by model type but also by the type of label, specifically, whether LLMs differ in their classification accuracy for unrelated, anti-immigration and pro-immigration human-annotated tweets. To test this, we developed a new model (Model 2), which extends Model 1 by including interaction terms between Model and Label (Eq. 4 in the SI Appendix).

**Fig. 3:**
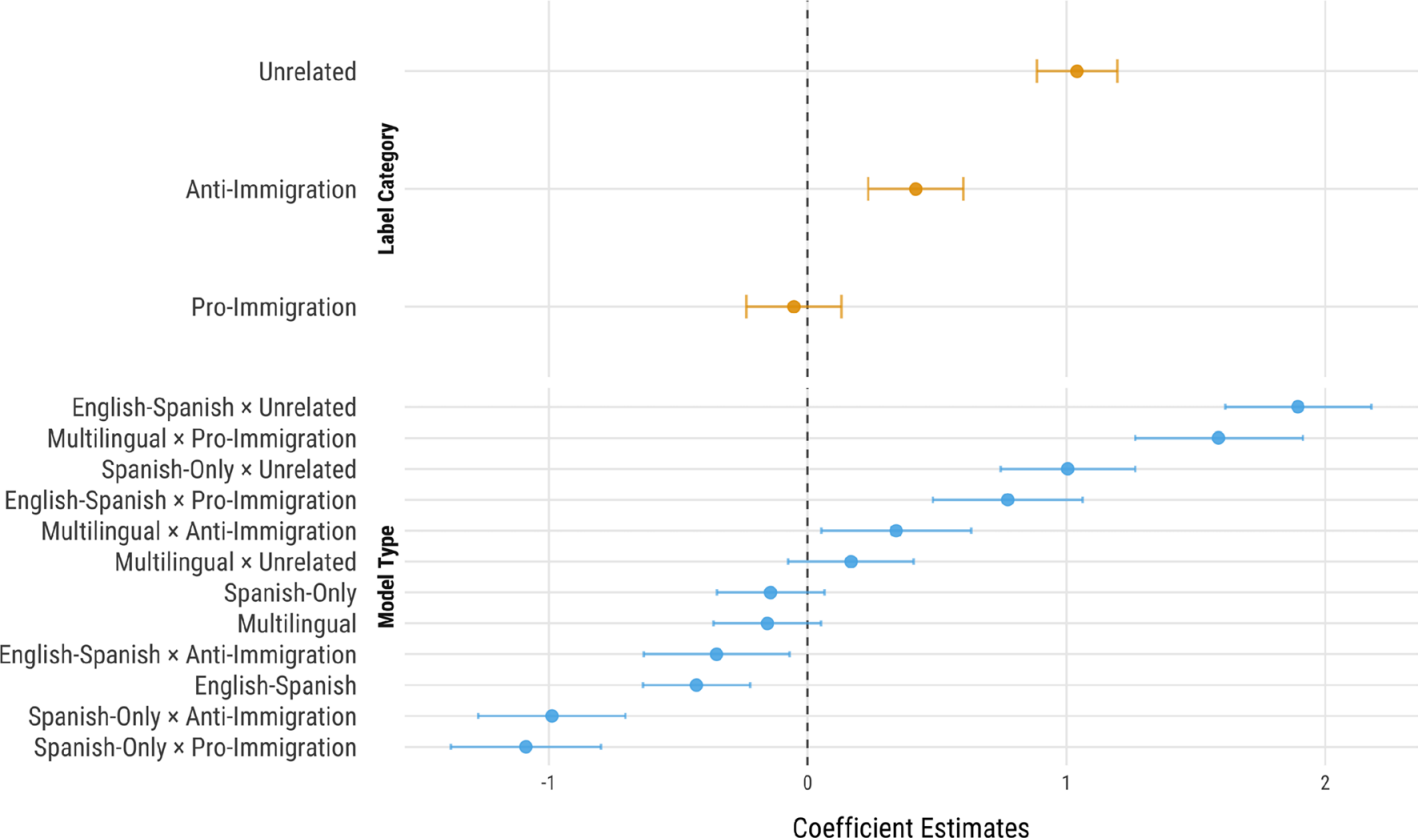
Estimated coefficients (log-odds) of model type and label category on classification accuracy. The figure shows coefficient estimates for *Model* and *Label* main effects and their interaction terms from Model 2 (Eq. 4 in the SI Appendix). The model estimated the probability that a fine-tuned language model correctly classifies a tweet’s stance toward immigration. The full model also includes controls for *Language*, *Translation Quality* and *Train/Test* split, which are not displayed in this figure. Estimates are presented in log-odds, with horizontal lines denoting 95% confidence intervals. The baseline category represents the English model applied to untranslated English tweets labeled as “neutral“ in the training set, with all coefficients showing effects relative to this reference point.

Figure 3 visualizes the estimated coefficients from Model 2, highlighting how each model performs across different label categories (see Table 11 in the SI Appendix for the full set of regression coefficients).

The results show a clear distinction in model strengths. The English-Spanish model performs significantly better ($\beta = 1.755$, $p < 2 \times 10^{-16}$) than the others at classifying unrelated content. This suggests that, even without being fine-tuned on multiple languages, the model is able to learn the general topic of immigration and distinguish unrelated tweets from immigration-related ones even in languages it was not exposed to during training.

In contrast, the Multilanguage model excels in detecting specific stances. It shows a strong positive effect in classifying both anti-immigration content ($\beta = 0.198$, $p = 0.084$) and especially pro-immigration content ($\beta = 1.622$, $p < 2 \times 10^{-16}$). These coefficients indicate that fine-tuning on multiple languages supports better sensitivity to subtle distinctions in sentiment or stance, even if overall topic understanding transfers across language boundaries.

Taken together, these results show that fine-tuning enables LLMs to learn the general topic of immigration regardless of the languages used during training. This is evident in the English-Spanish model’s strong performance in identifying unrelated content, even in languages it was not explicitly trained on. However, accurately detecting specific attitudes toward immigration, particularly pro-immigration stances, benefits from multilingual exposure, likely because the expression of ideological positions varies across languages.

These findings support the broader claim that while understanding whether content is about immigration is largely transferable across languages, capturing the nuances of ideological stance in multilingual data requires exposure to linguistic diversity during fine-tuning. This improvement reflects the combined effect of multilingual exposure and the increased training sample size that accompanies it, aligning with fundamental statistical principles that larger, well-curated training sets yield better model performance.

#### Accuracy of fine-tuned models varies by language

To investigate how classification performance varies across languages and model types, we estimated a logistic regression model (Model 3) with main effects for Model and Language, along with their interaction. This allows us to assess how each model performs in different linguistic settings while controlling for Label, Translation and Train/Test similarly to Model 1.

**Fig. 4:**
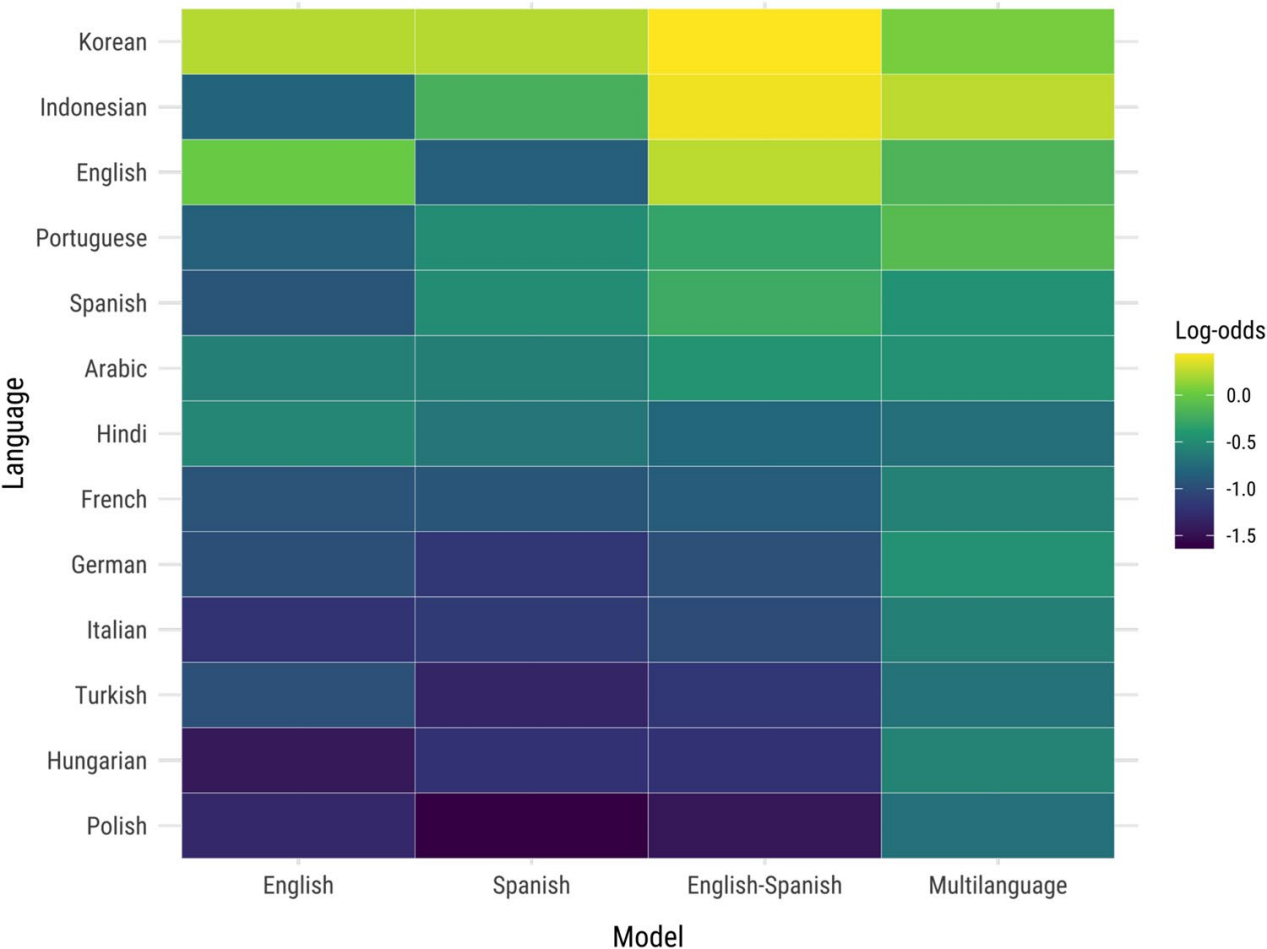
Cumulative log-odds of correct classification by model and language. Heatmap shows predicted classification accuracy for each combination of language on the y-axis and model on the x-axis (English, English-Spanish bilingual, Multilanguage and Spanish), based on coefficient estimates from Model 3 (Eq. 5 in the SI Appendix). Lighter cells indicate combinations where the model is more likely to classify tweets correctly, while darker cells indicate lower expected accuracy. For each cell, the total effect is computed as the sum of main and interaction terms involving the selected model and language, with all other covariates held constant.

Figure 4 displays a heatmap of log-odds for each combination of model and language based on estimates from Model 3. The visualization highlights substantial variation in model performance across linguistic contexts, derived from the main and interaction effects between fine-tuned model type and language. The full set of coefficient estimates from Model 3 is reported in the SI Appendix (Table 12).

We calculated total log-odds by summing the coefficients for each model, language and their interaction. Higher total values indicate a greater predicted probability of correct classification, whereas lower values indicate worse expected performance. We report the cumulative log-odds for each language and model in Table 12 in the SI Appendix.

The Multilanguage model performed better than other models in most languages, especially in Polish (–0.718) and Turkish (–0.573). This suggests that multilingual fine-tuning enabled the model to generalize better in challenging settings, even when classification difficulty remained high in absolute terms.

The English-Spanish model performed best in Indonesian (0.435) and Korean (0.545), the only two languages where any model achieved a positive total log-odds score. These results indicate that even without explicit exposure to these languages, the model successfully transferred topic understanding, likely due to structural or topical similarities in the training data.

The Spanish-only model underperformed across the board. Interestingly, even in Spanish, its total effect (–0.468) was more negative than that of the English-Spanish model (–0.272). This suggests that the English-Spanish model benefited from a larger training dataset and a more focused multilingual exposure, making it better suited than the Spanish-only model, even for classifying tweets in Spanish. We report the complete F1 scores in Table 6 in the SI Appendix.

These findings suggested that while the Multilanguage model generally achieved higher classification performance in languages seen during fine-tuning, the English-Spanish model performed particularly well in languages like Indonesian and Korean, suggesting that topic knowledge could transfer effectively across languages, even without explicit exposure.

#### Models fine-tuned in fewer languages are more accurate in identifying topic-relevant content

Label distributions varied notably across languages. For example, Korean tweets contained a substantially higher proportion of unrelated content than others which may complicate the interpretation of language-specific accuracy estimates. While Model 2 incorporated interactions between model and label, it did not address how differences in label distribution across languages might affect classification outcomes. In particular, previous models were unable to disentangle whether high performance in certain languages reflected true model understanding or simply an easier underlying label mix. Both Models 1 and 2 showed that unrelated tweets were generally easier to classify and Model 3 revealed variation in performance across language–model combinations. This raises an important issue: when a model performs well in a given language, is it due to its ability to interpret that language’s immigration discourse, or because the dataset contains a disproportionately high number of unrelated tweets? For example, the strong performance of the English–Spanish model on Korean tweets may not indicate a deep understanding of Korean-language immigration content, but rather reflect the overrepresentation of unrelated tweets in that subset. To address this limitation, we introduce a variable called ShareUnrelated, which captures the proportion of tweets labeled as unrelated to immigration within each language subset of our dataset. For example, if 70% of Korean tweets are classified by human annotators as unrelated, then every Korean-language observation is assigned ShareUnrelated = 0.70. This variable allows us to control for variation in label composition across languages and assess whether model performance is influenced by the relative ease of classifying unrelated content. We estimated a fourth logistic regression model (Model 4) that included the interaction term between ShareUnrelated and Model. This model builds on Model 3 by retaining the interaction between Model and Language, as well as Translation and Train/Test as control variables. By incorporating these additional factors, Model 4 allows for a more nuanced understanding of how both model architecture and dataset composition influence classification performance across languages. Table 13 in SI Appendix reports the complete log-odds from Model 4.

**Fig. 5:**
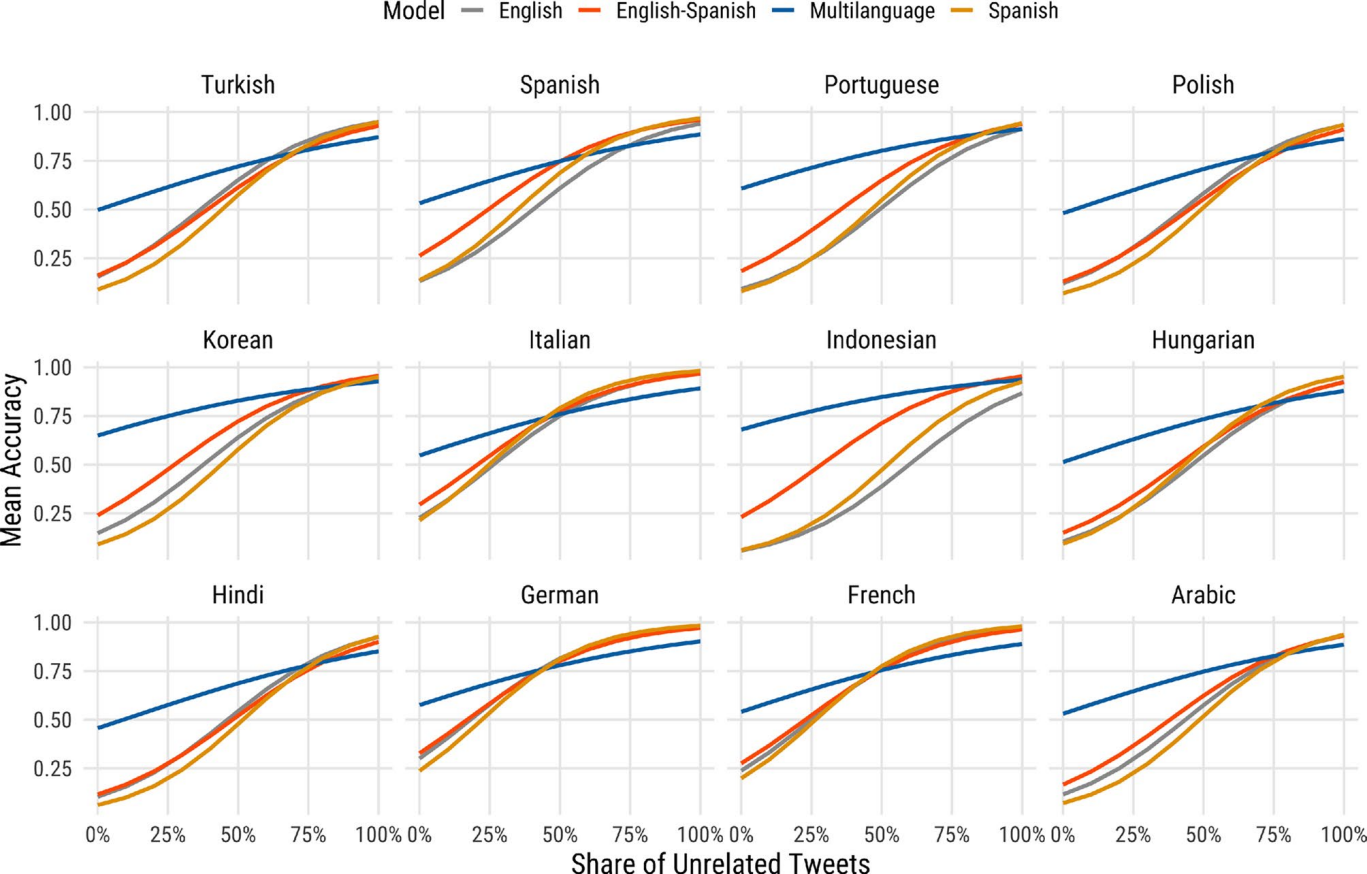
Predicted classification accuracy of fine-tuned language models as the share of unrelated tweets ranges from 0% to 100%. Accuracy curves are generated by inserting the estimated coefficients from the interaction model (Eq. 6 in the SI Appendix) into the logistic link function $\textrm{Accuracy}=1\big /\bigl (1+e^{-\textit{Total Effect}}\bigr )$, where *Total Effect* combines main and interaction terms for *Model*, *Language* and *Share Unrelated*. Lines correspond to four model variants (English, English–Spanish, Multilanguage and Spanish) and panels separate the language of the tweets being classified.

Figure 5 shows the compounded effect by summing three components: the Model’s main coefficient, the main effect of ShareUnrelated and the interaction term between Model and ShareUnrelated. The combined value represents the fine tuned model’s expected accuracy as the share of unrelated tweets changes. Higher values indicate a higher probability that the model correctly classifies tweets when a larger share of tweets in that language is unrelated to immigration. The English-Spanish model produced the highest compounded effect, with a total of 5.009 and all its contributing coefficients were statistically significant. The Spanish model followed with a total effect of 4.456, although its main effect was not statistically significant. The Multilanguage model had the lowest total effect at 3.597 and neither its model coefficient nor its interaction term was statistically significant. These findings suggest that in contexts where a large share of tweets are unrelated to immigration, the English and Spanish models tend to outperform the multilingual model. This may reflect a trade-off: while multilingual fine-tuning improves a model’s ability to detect ideological stance within immigration-related content, it may reduce its robustness when dealing with off-topic data. More broadly, the results show that models fine-tuned in only one or two languages can still learn to identify whether a tweet is about immigration. This suggests that the ability to recognize topic relevance can generalize across languages. In contrast, the multilingual model performs best when tweets are clearly about immigration, as it is more effective at distinguishing between nuanced stances. However, this strength may come at the cost of lower performance when processing unrelated or ambiguous content, where the English and Spanish models demonstrate greater reliability.

#### LLM language pretraining biases are associated with lower accuracy in specific languages

An important determinant of a fine-tuned LLM’s multilingual classification performance is the extent to which each language was represented in its original pretraining corpus. Since LLMs were trained predominantly on English texts^34^, we hypothesized that languages with greater presence during pretraining are likely to show higher classification accuracy. To examine this structural dimension, we rely on language share estimates from the LLaMA 2 pretraining corpus, as Meta has not publicly released detailed language composition data for the LLaMA 3 family of models which includes LLaMA 3.2 - 3B, the lightweight model that we have used for fine-tuning. While the exact figures for LLaMA 3 remain unknown, Meta has reported that only 8% of its pretraining tokens are non-English, closely aligning with the 11% reported for LLaMA 2. Given this similarity, we used the LLaMA 2 reported languages as a reasonable proxy to infer the likely distribution of language representation in LLaMA 3 models and to assess the potential impact of pretraining biases on model performance across languages. We assigned to each language in our dataset, the share of that language that is present in the pretraining corpus (see Table 5 in the SI Appendix for details).

**Fig. 6:**
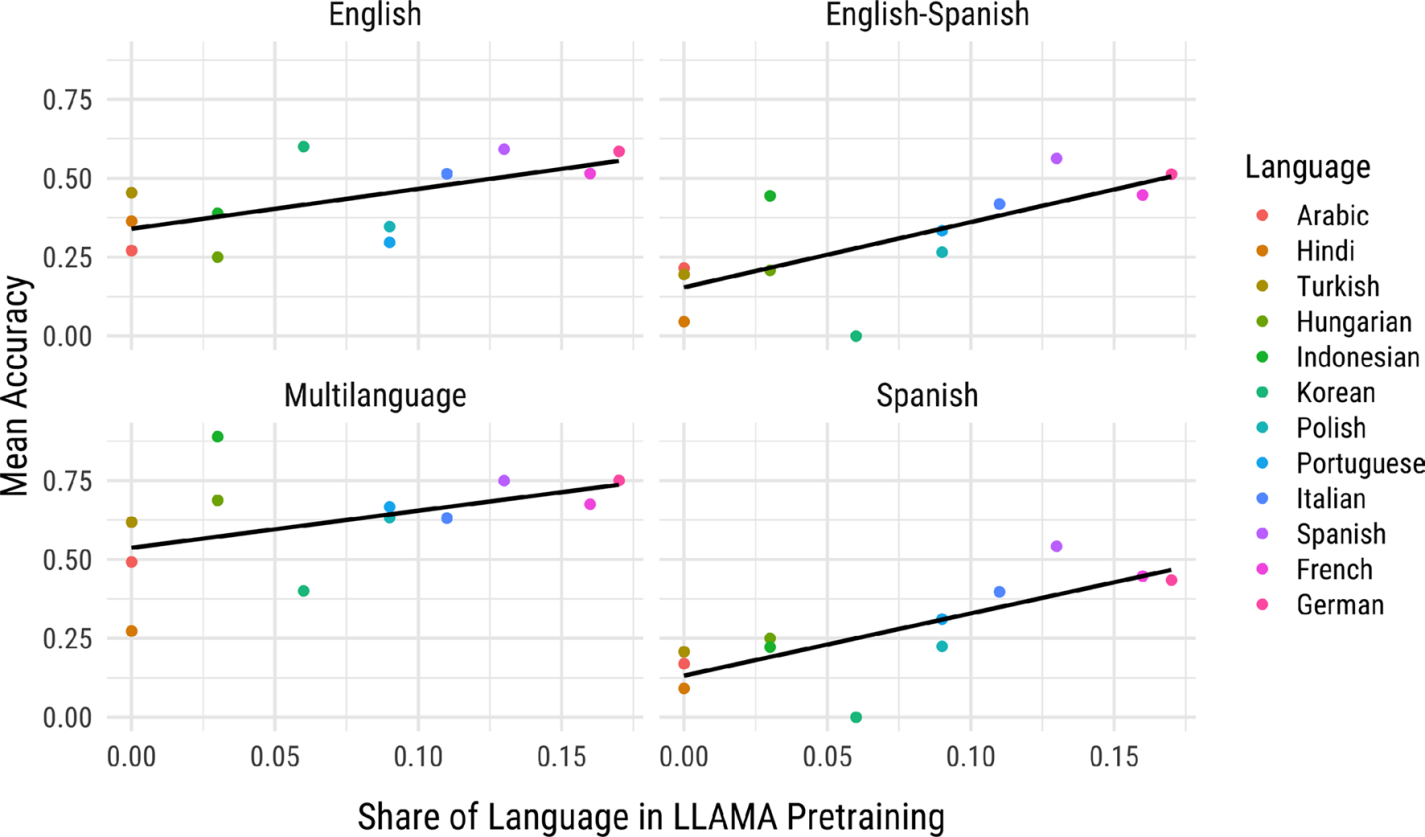
Classification accuracy increases with pretraining language exposure across all model variants. Each point represents mean accuracy for a language-model combination, plotted against the share of that language in the pretraining corpus. The black regression line shows the overall positive relationship between pretraining exposure and classification performance. Languages in the legend are ordered by the lowest share (eg. Arabic) in the LLaMA 2 corpus to the highest (eg. German).

Figure 6 illustrates how classification accuracy varies by model type, language and the percentage of language (except English) present in the pretraining corpus, as reported by Meta. To calculate the accuracy shown in the figure, we filtered out English tweets and tweets labeled as “unrelated“, grouped the data by language, model type and pretraining language share and computed the mean accuracy for each group based on the correct observations. The figure reveals that, for all LLMs, accuracy tends to be higher for languages with greater pretraining exposure. This pattern is particularly strong for the monolingual and bilingual models, suggesting that pretraining exposure influences accuracy across languages. This points to an underlying and potentially problematic bias in the text classification task based on the corpus of texts used for LLaMA’s pretraining. Our fine-tuned Multilanguage LLM displays a more stable level of classification accuracy even for languages with limited or no representation at the pretraining stage, such as Hungarian and Turkish. This may be explained by the Multilanguage LLM exposure during the fine-tuning phase to these under-represented languages which reduces the difference in accuracy generated by the language bias in the pretraining. These results suggested that introducing as few as 3000 tokens out of 15.6 trillion tokens of the pretraining, can meaningfully boost classification accuracy for that language and help counter the English-centric bias of today’s open- and closed-source LLMs.

#### Fine-tuned 3B model processes tweets 19–96$\times$ faster and 108–2017$\times$ cheaper than commercial LLM APIs such as GPT or DeepSeek

Beyond classification accuracy, our approach offers substantial advantages in terms of computational efficiency, cost and environmental impact. Table 8 in SI Appendix presents a comprehensive comparison of resource requirements for classifying 10 billion tweets using our fine-tuned Llama 3.2-3B model versus leading proprietary alternatives. All estimates are based on a common workload specification and should be interpreted as scenario-based comparisons rather than exact realized deployment costs. Specifically, the calculations assume a 76-token prompt, an average tweet length of 36 tokens and a 1 token output per classification. Our fine-tuned model, running on Harvard’s FASRC GPU cluster, achieves remarkable efficiency gains across multiple dimensions. Operating at 3,854 tokens per second, it processes text between 19 to 88 times faster than proprietary alternatives, with the speed advantage being most pronounced compared to DeepSeek models (96$$\times$$ faster than DeepSeek Chat, 79$$\times$$ faster than DeepSeek Reasoner) and substantial even against faster proprietary models like GPT-5.4 (46$$\times$$ faster). The cost implications are even more dramatic. We estimate that classifying the entire Harvard 10 billion Geotweet Archive 2.0 dataset would cost just $2,900 using our approach, compared to $313,000 for the least expensive proprietary alternative (DeepSeek Chat) and up to $5.85 million for Claude Opus 4.6. This represents cost reductions of 108$$\times$$ to 2017$$\times$$ compared to commercial APIs. For commercial models, these estimates are derived from public API prices per million input and output tokens, whereas for our fine-tuned model the estimate reflects inference electricity costs on Harvard’s FASRC infrastructure. Full details of the speed benchmarks, energy formulae, water and carbon intensity factors and API pricing sources are reported in the notes to Table 8 in SI Appendix.

Environmental benefits are equally significant. Our model, powered by Harvard’s renewable energy infrastructure, produces virtually zero carbon emissions, while proprietary alternatives generate between 271 to 3139 tonnes of CO_2_ for the same classification task. Water usage follows a similar pattern, with our approach requiring only 14.3 m^3^ compared to 347.7 to 37,750.4 m^3^ for commercial models^35^. These efficiency gains stem from several technical optimizations: (1) the use of a smaller, more efficient 3B parameter model compared to much larger proprietary alternatives (175B to 1.7T parameters), (2) 4-bit quantization that reduces model size by 50% while preserving accuracy, (3) LoRA fine-tuning that minimizes trainable parameters and (4) deployment on renewable energy infrastructure. Importantly, these optimizations do not compromise classification performance, as demonstrated by our accuracy results across languages and tasks. The resource efficiency of our approach addresses a critical barrier to multilingual LLM research: the assumption that high-quality performance requires either expensive proprietary models or computationally intensive training of separate models for each language. Our results demonstrate that a single, efficiently fine-tuned open-source model can achieve competitive multilingual performance while dramatically reducing financial, computational and environmental costs.

## Discussion

A central finding of this study is that fine-tuning large language models enables them to internalize topic understanding in a way that generalizes across languages and cultural contexts. Even when trained on data in just one or two languages, LLMs can learn to recognize whether content is topically relevant, in this case, whether a tweet is about immigration, including in languages the model has never seen during fine-tunining training. This suggests that topic understanding is not tied to specific linguistic patterns but reflects deeper semantic understanding that can transfer across multilingual environments. For example, our bilingual English–Spanish model performed well in identifying unrelated content across a range of typologically diverse languages, reinforcing the idea that topic comprehension is broadly transferable.

However, when the task shifts from recognizing topic relevance to classifying ideological stance, such as distinguishing between pro- and anti-immigration tweets. performance becomes more variable. The multilingual model, fine-tuned on a diverse set of twelve languages, consistently outperformed the monolingual and bilingual models in this more nuanced task, particularly in low-resource or typologically distant languages. Yet this improved sensitivity to ideological variation comes with a trade-off: the multilingual model is less accurate when classifying datasets with a high proportion of unrelated content. In such cases, simpler models trained on fewer languages, like the English–Spanish model, are more robust in filtering relevant from irrelevant content, likely because their training data offer a more focused signal. By accounting for the share of unrelated content in each language dataset, we observed that monolingual and bilingual models often outperformed the multilingual model when it came to identifying whether tweets were actually about immigration. This suggests a key trade-off: the multilingual model is more effective at detecting subtle differences in stance toward migration but less reliable at distinguishing relevant from irrelevant content, while simpler models are better at filtering off-topic tweets but less accurate in identifying ideological positions.

Our results suggest that imbalances in LLaMA’s pretraining language distribution influences its downstream performance. Languages with greater presence in the LLaMA pretraining corpus, such as German, French and Spanish, tended to show higher classification accuracy across all models, particularly the monolingual and bilingual ones. At the same time, our results do not allow us to attribute these cross language differences to pretraining alone. We nevertheless find that the multilingual model achieved relatively stable performance in languages such as Hungarian and Turkish, which are under represented or absent in the Llama pretraining. In several cases, exposure to as few as 75 annotated tweets, equivalent to equivalent to $8.65 \times 10^{-11}$ of the 15.6 trillion tokens used to pretrain LLaMA 3, was associated with visible gains in classification accuracy. These findings seem to suggest that targeted fine tuning may help narrow some cross language performance gaps, especially for lower resource languages. This pattern is consistent with prior work suggesting that multilingual model behavior reflects the composition of pretraining data and that targeted fine tuning can partially mitigate some resulting disparities, particularly in under represented languages and cross cultural settings^20, 22^.

Finally, our analysis highlights the cost of relying on translation as a strategy for multilingual classification. Tweets translated into English, regardless of the assessed translation quality, were classified less accurately than their original counterparts. Even “good” translations led to degraded model performance, suggesting that critical semantic or stylistic cues are lost in translation. This effect persisted even when using high-quality human-rated translations. These findings reinforce the importance of training models directly on native-language content rather than translating it into English. Exposure to the original linguistic and cultural framing appears necessary for models to fully capture the meaning of online discourse, particularly when it involves ideologically sensitive topics like immigration.

Together, these findings underscore both the promise and limits of multilingual generalization in fine-tuned LLMs. Topic-level understanding appears transferable even with minimal language exposure. However, for tasks requiring ideological nuance, multilingual fine-tuning is essential. It not only improves performance on seen languages but also enhances generalization to underrepresented and typologically distant ones. Crucially, this can be achieved even with very small, curated datasets, suggesting a practical path forward for improving equity in multilingual NLP and computational social science research.

## Conclusions

The rapid adoption of LLMs is transforming how social scientists analyze text across languages, scales and social domains. These tools promise efficiency and reach. Yet our understanding of how they function in multilingual, real-world settings remains limited, particularly in complex and culturally embedded issues like immigration. Much of the existing research relies on English-centric approaches, synthetic data and machine translation strategies that can obscure meaning and cultural nuance. Furthermore, existing studies often evaluate performance using standardized benchmarks that may not reflect the linguistic complexity and cultural specificity of real-world social science applications. Additionally, the field lacks systematic comparisons of monolingual versus multilingual fine-tuning approaches for culturally sensitive topics where linguistic and cultural context critically influence meaning. While fine-tuning separate monolingual models for each language appears to be one viable strategy for achieving optimal performance, this approach presents significant practical barriers for most social science research contexts: it requires multiple human-annotated datasets, substantial computational resources for training multiple models and considerably longer development timelines. These resource constraints may inadvertently limit multilingual research to well-funded institutions or force researchers to accept suboptimal performance across languages. The literature also provides limited guidance on how performance disparities across languages might compound existing inequalities in whose voices and perspectives are accurately captured in computational social science research. Moreover, while recent work has documented language representation imbalances in pretraining corpora, there has been limited empirical investigation of how these structural biases interact with fine-tuning strategies to shape performance across languages in domain-specific classification tasks. These knowledge gaps has significant implications for the design, deployment and ethical use of LLMs in the social sciences.

Our study addresses these gaps by examining how fine-tuning strategies affect multilingual classification performance in immigration discourse. We found that fine-tuned LLMs can successfully identify immigration-related content even in languages not seen during training, suggesting that topic-level knowledge generalizes across languages through the model’s deeper semantic representations. However, more nuanced tasks like stance detection–distinguishing supportive, opposing, or neutral positions–require richer linguistic exposure. These tasks rely on language-specific surface features such as morphology, orthography (eg. writing in capital letters) and cultural idioms (eg. “build the wall”) that vary significantly across languages. Consequently, multilingual fine-tuning substantially improves stance classification accuracy, particularly for underrepresented or typologically distant languages.

We also demonstrated that pretraining corpus composition creates systematic performance biases. Languages with greater representation in the original training data consistently achieved higher classification accuracy, though these gaps can be substantially reduced through targeted multilingual fine-tuning. We also found that the fine-tuned model labels tweets more accurately in their original language than in high-quality translations, indicating that translation introduces semantic noise.

We acknowledge some limitations. Our study focuses on immigration discourse and short-form social media content, as result, it should be interpreted in relation to this specific application and may not immediately generalize to other text classification tasks. Performances patterns observed here may not fully extend to longer texts outside social media, where linguistic structure and contextual information differ from short posts. Future research should examine whether these patterns hold across different domains and text types. Social media data present some biases. We did not remove content posted by automated accounts known as bots. However, content generated by bots are less than few share of the total volume of tweets in Harvard Geotweet Archive^36^ while previous studies have shown that bot-generated content is marginal in the Twitter/X debate on immigration^3^. While we identified associations between pretraining exposure and performance, further investigation is needed to establish causality and explore how cultural and discursive dimensions interact with fine-tuning strategies. Despite these limitations, our work provides actionable insights for building more inclusive and practical multilingual classification systems in computational social science.

Our findings offer both methodological and practical contributions. Methodologically, our work demonstrated that domain-specific knowledge can generalize beyond the training language, suggesting a scalable path forward for multilingual LLM applications without exhaustive data requirements. Practically, we showed that fine-tuning open-source LLMs using resource-efficient techniques like LoRA and 4-bit quantization offers a remarkably cost-effective and environmentally sustainable alternative to proprietary systems. Our fine-tuned Llama 3.2-3B model, running on Harvard’s FASRC GPU cluster, operates at 3,854 tokens per second–between 36 to 168 times faster than proprietary alternatives. We estimate that our approach could classify the entire Harvard 10 billion Geotweet Archive 2.0 dataset for just $2.9k with virtually zero carbon emissions, compared to proprietary alternatives that would cost between $316,000 and $17.7M while generating hundreds of tonnes of CO_2_. This lightweight framework enables scalable multilingual classification and dramatically lowers barriers to adoption for research teams with limited computational budgets, supporting broader efforts to democratize AI.

## Materials and methods

This study explores how fine-tuning a lightweight open-source LLM on human-annotated data improves its ability to classify tweets about immigration across multiple languages. Our approach combines three main components: the collection and annotation of multilingual tweets, the fine-tuning of models with different levels of language exposure and a structured evaluation of classification performance across topic and stance. Immigration serves as an ideal test case due to its global relevance, ideological complexity and prominence in online discourse, where it often triggers polarized debate and targeted hostility. By using human annotations to validate model predictions, we assess whether fine-tuning enables LLMs to learn the concept of immigration in a way that generalizes across linguistic and cultural boundaries.

### Open source LLMs: LLaMA 3.2, 3B parameters and multilanguage BERT

We built several models to classify immigration-related content in multilanguage tweets, with the goal of deploying them on very large-scale social media data. This scale required a model that is both efficient and scalable. We fine-tuned the LLaMA 3.2 model with 3 billion parameters, an open-source model released by Meta AI in 2024. In addition, we fine-tuned a Multilanguage BERT (mBERT) model to serve as an encoder-based baseline for comparison.

We selected LLaMA over commercial alternatives such as ChatGPT (OpenAI) or Claude (Anthropic) for several reasons. First, open-source models like LLaMA offer full access to model weights and architecture, enabling fine-tuning and modification at all levels, an essential feature for replicable research and understanding how models learn. In contrast, proprietary models limit fine-tuning to constrained API-based methods and do not support retraining of core model layers. Second, using closed APIs at this scale would have been prohibitively expensive, with inference alone estimated between $237,000 and over $5.8 million (see Table 8 in the SI Appendix). By combining an open-source model with university cluster infrastructure, we dramatically reduced the cost of training and inference. Third, using an open-source model allowed us to release the resulting classifier publicly, supporting open science and enabling reuse in other domains. Finally, we optimized the model for deployment by converting it into GGUF format and applying 4-bit quantization, which reduced memory usage and inference time with minimal impact on accuracy. To measure whether quantization materially altered classification performance, we compared the F1 scores of the unquantized and 4-bit quantized versions of each fine-tuned model across the main evaluation datasets. As shown in Table 9 in SI Appendix, the average percentage difference in F1 between the unquantized and quantized variants ranged from 1.6% to 4.0% across models, indicating that 4-bit quantization had only a limited effect on classification performance in our setting.

Among the open-source options available at the time of processing (Fall 2025), LLaMA 3.2 offered one of the strongest multilanguage foundations, having been trained on 15.6 trillion tokens across multiple languages, an order of magnitude more than earlier versions like LLaMA 2, which was trained on only 1.5 trillion tokens. We selected the 3 billion parameter version to balance predictive performance with practical constraints around speed, memory and deployment cost. As shown in Carammia et al. (2024)^13^, small fine-tuned models can perform as well as or better than much larger base models from the same family or across model families. For example, a fine-tuned LLaMA 3 model with 3 billion parameters outperforms a 70 billion parameter base model, while being significantly more efficient in terms of inference speed and memory usage. Moreover, the marginal performance gains from fine-tuning very large models tend to diminish, making smaller models more attractive for large-scale classification. These combined factors made LLaMA 3.2, 3B a robust and scalable solution for multilanguage tweet classification at scale.

Multilanguage BERT (mBERT) represents a widely used encoder-only baseline in cross-lingual classification tasks. The model contains approximately 110 million parameters and is based on the BERT-base architecture introduced by^6^. It was pretrained using a masked language modeling objective on concatenated Wikipedia corpora covering more than 100 languages without explicit cross-lingual alignment. Its bidirectional transformer architecture enables the encoding of contextual representations across languages, while its relatively small parameter scale makes it computationally efficient and suitable for large-scale deployment. To ensure a controlled comparison, we fine-tuned mBERT on the same human-annotated dataset as LLaMA using identical train-test splits and classification objectives, allowing us to isolate the effect of model architecture and pretraining scale on downstream performance.

The substantial differences in both pretraining scale and model architecture make newer LLMs qualitatively different from BERT, limiting the degree to which insights from earlier work can be extended to LLaMA-style models. Notably, prior studies have found that LLaMA models can outperform BERT-based transformers in multilanguage hate speech classification, particularly for implicit or indirect abuse, a feature that the online immigration debate is especially prone to^12^. However, this advantage has not been systematically tested for classifying immigration-related content across multiple languages and at scale.

Taken together, this experimental design enables a direct comparison between encoder-based and decoder-only architectures, allowing us to assess whether differences in pretraining scale and model structure translate into meaningful performance differences in multilanguage topic and stance classification.

### Fine-tuning: LoRa

Fine-tuning allows us to specialize LLMs for specific tasks without retraining from scratch. We fine-tuned the LLaMA models using Low-Rank Adaptation (LoRA), which modifies the model’s output to better suit our particular classification task. Specifically, we fine-tuned Meta Llama 3.2 3B Instruct in 4-bit quantized form using LoRA with rank $r=64$, $\alpha =32$, and dropout $=0.1$, trained for 10 epochs with a learning rate of $2\times 10^{-4}$, batch size $=16$, maximum sequence length $=1024$, constant learning rate scheduling, gradient checkpointing, and the paged AdamW 32-bit optimizer. Similarly, we fine-tuned a encoder-only multilingual BERT using the same training setup for LLaMA model except the quantization. Unlike proprietary models, such as Anthropic’s Claude, open-source fine-tuned models can be freely shared, advancing reproducible (social) science.

For fine-tuning LLaMA model, we used Harvard’s Faculty of Arts & Sciences Research Computing (FASRC) high performance computer cluster including A100 and H100 GPUs. For the classification tasks we used A100 and H100 GPUs. Fine-tuning and classification were performed using spare cycles of the cluster (i.e. when GPUs where idling). This required optimized strategies of check-pointing both fine-tuning and inference tasks as our processes could get terminated by legitimate GPU owners at any time during the execution. In this way, our project did not generate additional CO2 compared to the standard usage of an existing cluster^35^.

### Human annotation and inter-annotator agreement

Because supervised fine-tuning requires labeled examples, we first constructed a multilingual annotated dataset to train and evaluate the models. Tweets were independently labeled by two annotators per language. For most languages, both annotators were native speakers of the relevant language. For Indonesian, Hungarian, Korean and Arabic, one annotator was a native speaker while the second annotator worked from machine translations produced by Google Translate, given the practical difficulty of recruiting two qualified native speakers for these languages. This translation support was used exclusively during the annotation process and played no role in the classification pipeline evaluated in this study. Annotators were trained using a shared codebook defining the four label categories (unrelated, neutral, pro-immigration and anti-immigration), with worked examples and explicit guidance on boundary cases. Some annotators were also expert on immigration which further enhanced the overall labeling process. Annotation guidelines were developed iteratively across pilot rounds, with edge cases discussed and resolved before the main annotation began. For cases where annotators were uncertain or disagreed, each was asked to leave a written comment describing the content of the tweet and their interpretive reasoning. These comments were reviewed by the authors, who took the final labeling decision. This adjudication step was particularly important for tweets involving coded language, irony, or culturally specific references, which are especially common in immigration discourse. Overall inter-annotator agreement was 66%, corresponding to a Cohen’s kappa of $\kappa = 0.47$, calculated from the observed agreement and the expected chance agreement derived from the empirical label distribution in the dataset (53% unrelated, 17% anti-immigration, 16% pro-immigration, 15% neutral). This is consistent with the broader literature on the annotation of hateful speech content, where kappa values vary considerably depending on annotator expertise and task complexity, ranging from $\kappa = 0.57$ among amateur annotators to $\kappa = 0.84$ among expert annotators in comparable Twitter annotation tasks^37^^38^.

### Translation

To assess the impact of translation within a multilingual classification pipeline, we employed a 4-bit quantized version of the LLaMA 3.2 3B Instruct model to translate tweets from non-English languages into English. While larger models (eg. LLaMA 3.1 70B) may offer higher translation fidelity, their resource demands make them impractical for large-scale use since they would require extensive GPU infrastructure, which is inconsistent with our lightweight and reproducible inference pipeline. Similarly, commercial LLM APIs would require massive financial resources with an estimated cost around $2.9 million just for translating 10 billion tweets using the Open AI GPT 5.4 model. As result, our translation approach ensures consistency with the speed, computational and financial constraints of our broader evaluation workflow.

### Datasets

Our analysis draws on data from the Harvard Geotweet Archive 2.0^36^, a large-scale collection of over 10 billion geolocated tweets. To our knowledge, this is by far the largest publicly available social media dataset, more than five times the size of the 2 billion tweet dataset developed by Imran et al.^39^. This scale provides a unique opportunity to study multilanguage online discourse at an unprecedented level of granularity and coverage. From this dataset, we extracted and labelled sample of data in 13 languages. These data were used to fine-tune the models and test their performances.

To build the logistic regression models, we constructed a dataset by aggregating classification outcomes from four fine-tuned models: English-only, Spanish-only, English-Spanish bilingual and a Multilanguage model. Each of these LLMs classified all 4,900 human-annotated tweets. Additionally, the English-only LLM classified 1,100 non-English tweets that were machine-translated to measure the impact of translation on classification accuracy (see Table 3 in the SI Appendix).

Each observation in our dataset has the following variables:

- Tweet language (e.g., English, Spanish, Arabic),
- Tweet label (pro-immigration, anti-immigration, neutral, or unrelated),
- Translation quality, based on human assessment (good, bad, unknown, or not translated),
- Model used to classify the tweet, indicating the language(s) it was fine-tuned on,
- Dataset split, denoting whether the observation came from the training or test set.

The binary dependent variable was coded as 1 if the predicted label matched the human-assigned label and 0 otherwise. Additionally, we assigned to each tweet Language the reported proportion of that language in the pretraining corpus of LLaMA 2, the foundational open-source model used in the fine-tuning. To estimate language coverage in pretraining, we relied on publicly reported statistics for LLaMA 2 since granular data for LLaMA 3 have not been released. Meta reports that approximately 8% of its pretraining tokens are in non-English languages, while LLaMA 2’s pretraining corpus contains about 10.3% non-English content. While the specific language distributions may differ between the two models, this comparison provides a reasonable proxy for estimating language exposure. The language distribution from LLaMA 2’s pretraining corpus is reported in Table 5 in the SI Appendix.

### Datasets

To build the logistic regression models, we constructed the dataset by aggregating classification outcomes from four fine-tuned models: English-only, Spanish-only, English-Spanish bilingual and a Multilanguage model. Each of these LLM have classified all the 4,900 human-annotated tweets. Additionally, the English-only LLM classified 1100 non-English tweets which were machine-transalted to measure the impact of the translation on the accuracy outcomes (see Table 3 in the SI Appendix).

Each observation in our dataset has the following variables:

- Tweet language (e.g., English, Spanish, Arabic),
- Tweet label (pro-immigration, anti-immigration, neutral, or unrelated),
- Translation quality, based on human assessment (good, bad, unknown, or not translated),
- Model used to classify the tweet, indicating the language(s) it was fine-tuned on,
- Dataset split, denoting whether the observation came from the training or test set.

The binary dependent variable was coded as 1 if the predicted label matched the human-assigned label and 0 otherwise. Additionally, we have assigned to each tweet Language, the reported proportion of that language in the pretraining corpus of LLaMA 2, the foundational open source model used in the fine-tuning. To estimate language coverage in pretraining, we relied on publicly reported statistics for LLaMA 2 since granular data for LLaMA 3 have not been released. Meta reports that approximately 8% of its pretraining tokens are in non-English languages (Meta, 2024). In contrast, LLaMA 2’s pretraining corpus contains about 10.3% non-English content. While the specific language distributions may differ between the two models, this comparison provides a reasonable proxy for estimating language exposure. The language distribution from LLaMA 2’s pretraining corpus is reported in Table 5 in SI Appendix.

### Model specifications

To assess model performance across different linguistic and experimental conditions, we fit a series of logistic regression models predicting the probability that a tweet is classified correctly. These models include several predictors such as model type, language, label category, translation status and if a tweet belongs to train or test set, along with key interaction terms (e.g., Model $\times$ Language, Model $\times$ Label). All models are estimated at the tweet level and include robust standard errors. Full model formulas, coefficient tables and additional robustness checks are provided in SI Appendix.

## Supplementary Information

Supplementary Information.

## Data Availability

Data can be accessed: https://github.com/andreanasuto/immigration-llm/tree/main/model_performance/data.

## Code availability

Code can be accessed at: https://github.com/andreanasuto/immigration-llm.

## References

[1] Le Mens, G. & Gallego, A. Positioning political texts with large language models by asking and averaging. *Polit. Anal.* 1–9 (2025).

[2] Loukas, L., Stogiannidis, I., Diamantopoulos, O., Malakasiotis, P. & Vassos, S. Making llms worth every penny: Resource-limited text classification in banking. In *ICAIF 2023 - 4th ACM International Conference on AI in Finance*. Vol. 1(11). 392–400 (2023).

[3] Nasuto, A. & Rowe, F. Understanding anti-immigration sentiment spreading on Twitter. *PLOS ONE* **19**(e0307917), 9 (2024). 10.1371/journal.pone.0307917 PMC11373840 39231099

[4] Iacus, S.M., Qi, H. & Han, J. Deep Literature Reviews: An Application of Fine-Tuned Language Models to Migration Research. Vol. 4 (2025).

[5] Gilardi, F., Alizadeh, M. & Kubli, M. Chatgpt Outperforms Crowd Workers for Text-Annotation Tasks. Vol. 120 (2023). 10.1073/pnas.2305016120 PMC10372638 37463210

[6] Devlin, J., Chang, M.W., Lee, K. & Toutanova, K. Bert: Pre-training of deep bidirectional transformers for language understanding. In *NAACL HLT 2019 - 2019 Conference of the North American Chapter of the Association for Computational Linguistics: Human Language Technologies - Proceedings of the Conference*. Vol. 1(10). 4171–4186 (2018).

[7] Albladi, A. et al. Hate speech detection using large language models: A comprehensive review. *IEEE Access* **13**, 20871–20892 (2025).

[8] Pires, T., Schlinger, E. & Garrette, D. How multilingual is multilingual bert? In *ACL 2019 - 57th Annual Meeting of the Association for Computational Linguistics, Proceedings of the Conference*. 4996–5001 (2019).

[9] Zhang, J., Huang, Y., Liu, S., Gao, Y. & Hu, X. Do Bert-Like Bidirectional Models Still Perform Better on Text Classification in the Era of LLMS? Vol. 11. 18980–18989 (2025).

[10] Meta AI. *The llama 3 Herd of Models* (2024).

[11] Wei, J. et al. Emergent abilities of large language models. In *Transactions on Machine Learning Research* (2022).

[12] Singh, D.D., Bhattacharjee, R. & Chakraborty, A. *Rethinking Hate Speech Detection on Social Media: Can LLMS Replace Traditional Models?* Vol. 6 (2025).

[13] Carammia, M., Iacus, S.M. & Porro, G. Rethinking Scale: The Efficacy of Fine-Tuned Open-Source LLMS in Large-Scale Reproducible Social Science Research. Vol. 10 (2024).

[14] Wang, R. et al. Optimizing Large Language Model Training Using fp4 Quantization. Vol. 1 (2025).

[15] Bizzoni, Y. et al. How Human is Machine Translationese? Comparing Human and Machine Translations of Text and Speech. 280–290.

[16] Ji, M., Bouillon, P. & Seligman, M. Cultural and linguistic bias of neural machine translation technology. In *Translation Technology in Accessible Health Communication*. Vol. 8. 100–128 (2023).

[17] Savoldi, B., Gaido, M., Bentivogli, L., Negri, M. & Turchi, M. Gender bias in machine translation. In *Transactions of the Association for Computational Linguistics*. Vol. 9(8). 845–874 (2021).

[18] Zhao, Y. et al. Deciphering the Impact of Pretraining Data on Large Language Models Through Machine Unlearning. 9386–9406.

[19] Alkhamissi, B., Elnokrashy, M., Alkhamissi, M. & Diab, M. Investigating Cultural Alignment of Large Language Models.

[20] Huo, W. et al. Enhancing Non-English Capabilities of English-Centric Large Language Models Through Deep Supervision Fine-Tuning. (2025).

[21] Tao, Y., Viberg, O., Baker, R.S. & Kizilcec, R.F. Cultural bias and cultural alignment of large language models. *PNAS Nexus* **3**(9), pgae346 (2024). 10.1093/pnasnexus/pgae346 PMC11407280 39290441

[22] Ramezani, A. & Xu, Y. The discordance between embedded ethics and cultural inference in large language models. In *Proceedings of the 2025 Conference on Empirical Methods in Natural Language Processing* (Christodoulopoulos, C., Chakraborty, T. et al. eds.). 14715–14736 (Association for Computational Linguistics, 2025).

[23] Alesina, A. & Tabellini, M. The political effects of immigration: Culture or economics? *J. Econ. Lit.* **62**(3), 5–46 (2024).

[24] Bursztyn, L., Egorov, G., Enikolopov, R. & Petrova, M. Social media and xenophobia: Evidence from Russia. In Technical Report. Vol. 12 (National Bureau of Economic Research, 2019).

[25] Liu, Z. et al. Can LLMS Grasp Implicit Cultural Values? Benchmarking LLMS’ Cultural Intelligence with CQ-Bench. Vol. 4 (2025).

[26] Ahuja, K. et al. MEGA: Multilingual Evaluation of Generative AI. Vol. 10 (2023).

[27] Whitehouse, C., Choudhury, M. & Aji, A.F. LLM-powered data augmentation for enhanced cross-lingual performance. In *EMNLP 2023 - 2023 Conference on Empirical Methods in Natural Language Processing, Proceedings*. Vol. 5. 671–686 (2023).

[28] Castillo-López, G., Riabi, A. & Seddah, D. Analyzing Zero-Shot Transfer Scenarios Across Spanish Variants for Hate Speech Detection. 1–13 (2023).

[29] Lewis, B. & Jain, D. Harvard CGA Geotweet Archive v2.0. Dataset (2016).

[30] Artetxe, M., Goswami, V., Bhosale, S., Fan, A. & Zettlemoyer, L. Revisiting machine translation for cross-lingual classification. In *EMNLP 2023 - 2023 Conference on Empirical Methods in Natural Language Processing, Proceedings*. 6489–6499 (2023).

[31] Lai, V.D. et al. ChatGPT beyond English: Towards a comprehensive evaluation of large language models in multilingual learning. In *Findings of the Association for Computational Linguistics: EMNLP 2023*. 13171–13189 (2023).

[32] Zhang, S. et al. Getting more from less: Large language models are good spontaneous multilingual learners. In *EMNLP 2024 - 2024 Conference on Empirical Methods in Natural Language Processing, Proceedings of the Conference*. 8037–8051 (2024).

[33] Hu, E. et al. Lora: Low-rank adaptation of large language models. In *ICLR 2022 - 10th International Conference on Learning Representations*. Vol. 6 (2021).

[34] Guo, Y. et al. Do Large Language Models Have an English Accent? Evaluating and Improving the Naturalness of Multilingual LLMS.

[35] Jegham, N. et al. *How Hungry is AI? Benchmarking Energy, Water, and Carbon Footprint of LLM Inference*. Vol. 5 (2025).

[36] Lewis, B. & Kakkar, D. *Harvard CGA Geotweet Archive v2.0* (2016).

[37] Waseem, Z. & Hovy, D. Hateful symbols or hateful people? Predictive features for hate speech detection on twitter. In *HLT-NAACL 2016 - 2016 Conference of the North American Chapter of the Association for Computational Linguistics: Human Language Technologies, Proceedings of the Student Research Workshop*. 88–93 (2016).

[38] Waseem, Z. Are you a racist or am I seeing things? Annotator influence on hate speech detection on Twitter. In *NLP + CSS 2016 - EMNLP 2016 Workshop on Natural Language Processing and Computational Social Science, Proceedings of the Workshop*. 138–142 (2016).

[39] Imran, M., Qazi, U. & Ofli, F. Tbcov: Two billion multilingual COVID-19 tweets with sentiment, entity, geo, and gender labels. *Data* **7**, 1 (2022).

